# The Many Faces of Osteomyelitis: A Pictorial Review

**DOI:** 10.5334/jbr-btr.1300

**Published:** 2017-05-11

**Authors:** Julie Desimpel, Magdalena Posadzy, Filip Vanhoenacker

**Affiliations:** 1 Department of Radiology, Antwerp University Hospital, Edegem, BE; 2 Department of Radiology, AZ Sint-Maarten, Duffel-Mechelen, BE; 3 Department of Radiology, W. Dega Orthopaedic and Rehabilitation University, Karol Marcinkowski University of Medical Sciences, Poznan, PL; 4 Faculty of Medicine and Health sciences, Ghent university, Ghent, BE

**Keywords:** Osteomyelitis, Plain radiography, CT, MRI, Ultrasound

## Abstract

The purpose of this pictorial review is to present an overview of the radioclinical features of osteomyelitis.

The presentation of the disease may vary depending on the clinical stage (acute, subacute and chronic), the pathogenesis of the infection and the age of the patient. Thorough knowledge of the basic pathophysiological mechanisms is a prerequisite to understanding the variable imaging appearance of osteomyelitis.

Special subtypes of osteomyelitis including CRMO and SAPHO will be discussed very shortly.

## Introduction

Osteomyelitis (OM) is defined as an infection of the bone marrow and adjacent osseous structures with potential surrounding soft tissue extent. OM has a variable imaging appearance and therefore often mimics other bone diseases [1]. The purpose of this pictorial review is to discuss the different clinical and imaging features of OM, with a special emphasis on those helping to establish a confident diagnosis.

## General Clinical Presentation

Generally one may distinguish three clinical stages (acute, subacute and chronic), although in clinical practice there may be some overlap between these stages.

The routes of contaminations may vary in those clinical stages. In children hematogenous spread is the predominant route of infection, whereas in adults spread from a contiguous source, direct contamination or post-operative infection is much more frequent.

Furthermore, clinical manifestations may also differ according to the age of the patients. Particularly in infants the clinical findings are often more pronounced including local swelling, pain, reduced movement or refusal to move the affected limb, particularly in the acute phase. On the contrary, in adults the clinical onset is often more insidious. In children the tubular bones such as tibia and femur are the most common sites of infection, whereas the axial skeleton is most frequently affected in adults.

Laboratory findings typically show an increase of c-reactive protein (CRP) and erythrocyte sedimentation rate (ESR) especially in acute osteomyelitis in children whilst the white blood count may be normal. The evolution of the CRP levels correlate with the response to the therapy. Cultures are essential for accurate treatment but in acute setting only half of the blood cultures are positive impeding the diagnosis [2,3,4].

## Pathogenesis and Classification

**Figure 1:**
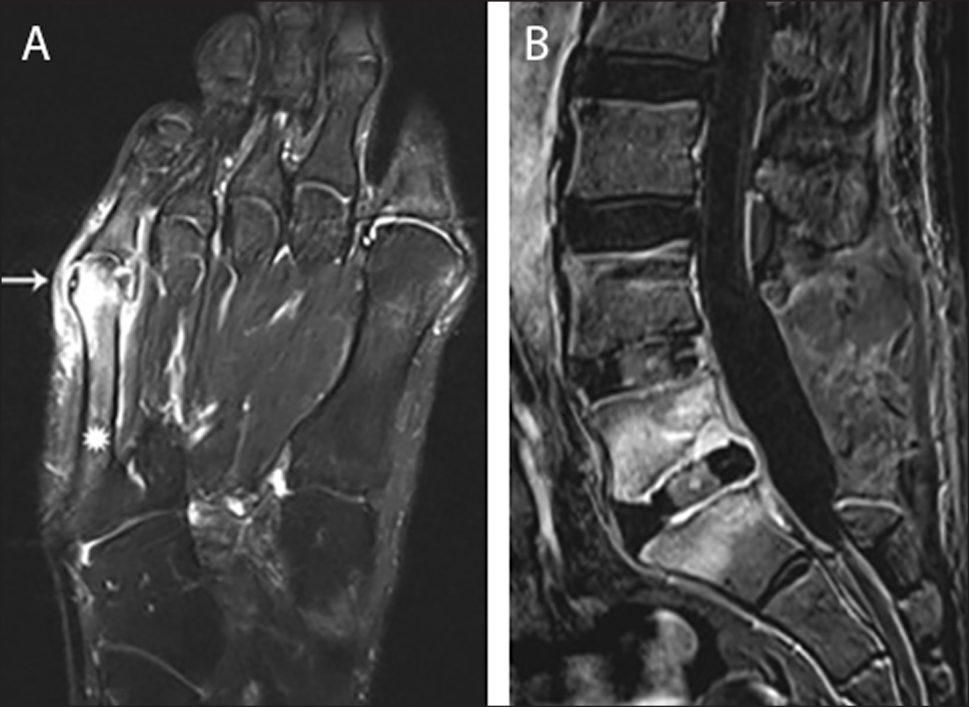
**Main routes of infectious spread. (a)** Infectious spread from contiguous infected tailors bunion. Axial T1-weighted image (WI) with Fat Suppression (Fat–Sat) after gadolinium contrast administration shows an inflamed bunion (white arrow) with adjacent enhancement of the distal aspect of the 5^th^ metatarsal (asterisks). **(b)** Postoperative infectious spread. Postoperative spinal infection. Sagittal T1–WI with Fat–Sat after gadolinium contrast administration. Note laminectomy L4 and L5 with placement of intervertebral cages. Extensive enhancement of L5 and S1 with rim enhancement of the intervertebral cage L5–S1 in keeping with postoperative spondylodiscitis.

There are different routes of contamination of which hematogenous spread of a micro-organism is the most frequent. In 95 percent of the cases the micro-organism is Staphylococcus aureus, but a variety of micro-organisms may be responsible. [2] Other potential routes of contamination consist of spread from a contiguous source of infection (Figure 1a), open fractures with direct implantation and/or presence of a foreign body or post-operative infection due to instrumentation (Figure 1b). Chronic OM is also associated with vascular insufficiency (e.g. due to underlying diabetes mellitus) leading to chronic wounds. This manuscript will mainly focus on hematogeneous spread of osteomyelitis. Further detailed discussion of the other pathways of spread is beyond the scope of this pictorial review.

**Figure 2:**
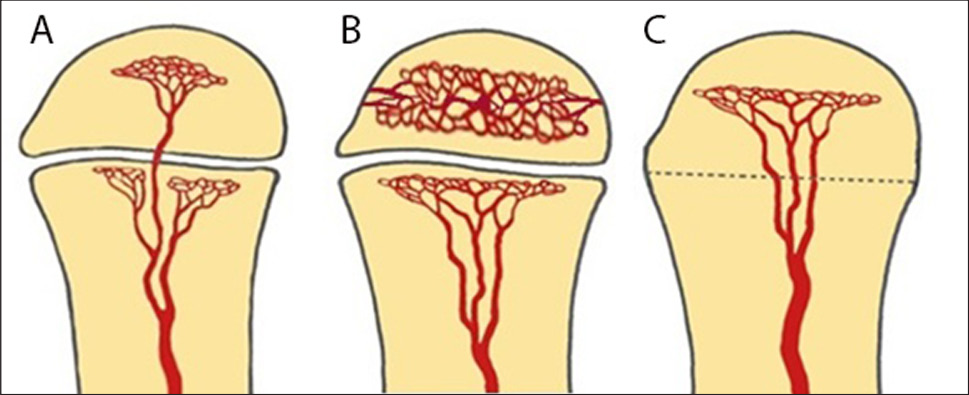
Schematic drawing of vascularization of the long bone, modified from Resnick. [2]. **(a)** Schematic drawing of vascularization of the long bone in infants younger than 18 months. There are metaphyseal and transphyseal blood vessels allowing metaphyseal and epiphyseal origin of infection. **(b)** Schematic drawing of vascularization of the long bone in children between 18 months and 16 years. The epiphysis has its own nutrient vessels (veins and arteries) whilst the metaphysis and diaphysis share the same vessels. A natural barrier is formed by the physis preventing spread of osteomyelitis in the epiphysis and joints. Therefore, children of this age group will present with an initial and predominant metaphyseal focus of infection. **(c)** Schematic drawing of vascularization of the long bone in adults after closure of the growth plate. From 16 years on restoration of the transphyseal vascularization may cause potential epiphyseal spread of infection.

The nature of blood supply to the diaphysis, metaphysis and epiphysis depends on the age of the patient. Thorough knowledge of these different patterns allows an understanding of the different radiological patterns of OM between children and adults (Figure 2) [5].

**Figure 3:**
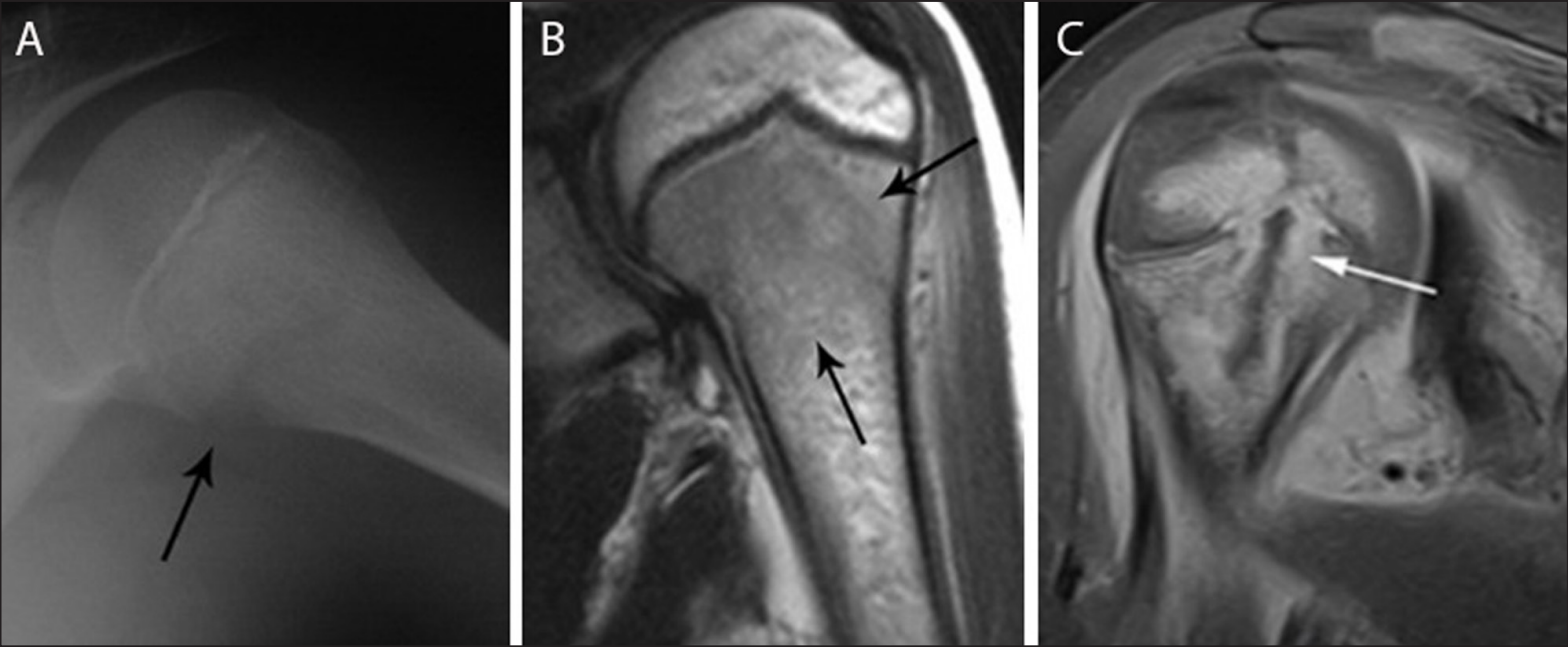
Acute osteomyelitis in 2 different patients. Plain radiograph **(a)** and coronal T2–WI **(b)** of acute osteomyelitis in the left proximal humerus. Another example of a child with osteomyelitis in the right proximal humerus **(c)** on coronal T1–WI with FS after gadolinium contrast administration. Standard radiography **(a)** shows a subtle osteolytic lesion at the metaphysis and loss of cortical delineation of the medial humerus (black arrow). The surrounding bone marrow edema (black arrows) **(b)** is restricted to the metaphysis in most cases of childhood osteomyelitis. The relativity of the barrier of the growth plate on MRI is illustrated in **(c)** Although a rim-enhancing intra-osseous abscess (white arrow) corresponding with the main site of infection is located at the metaphysis, there is also focal enhancement of the growth plate and epiphysis, in keeping with metaphyseal crossing.

**Figure 4:**
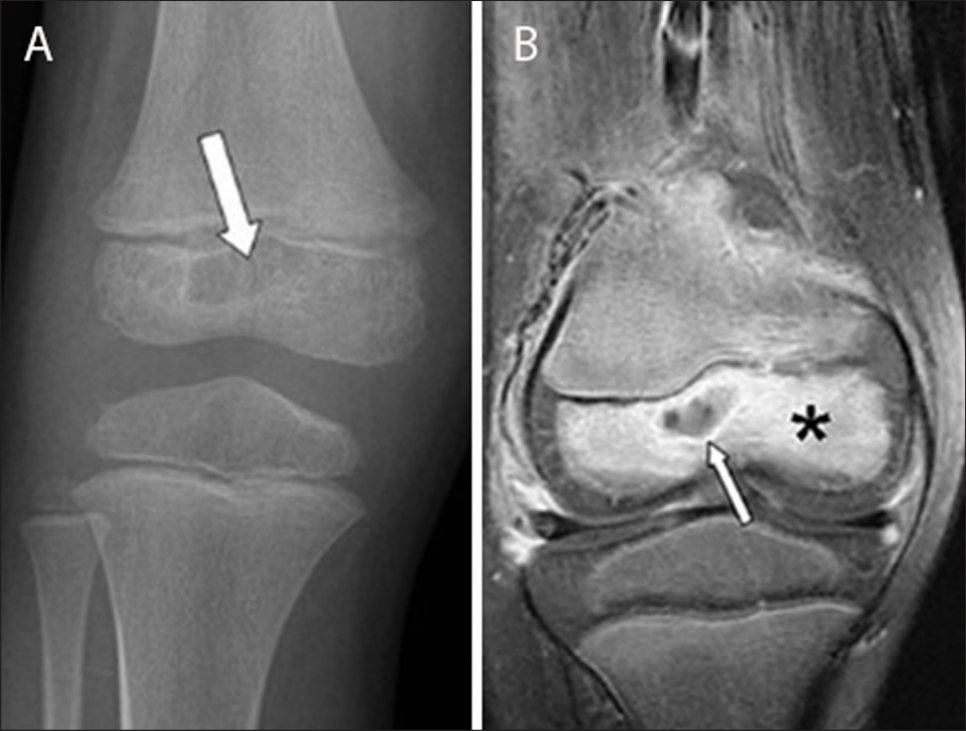
Epiphyseal childhood osteomyelitis of the right knee. Plain radiograph **(a)** of the distal femur shows a radiolucent lesion with peripheral sclerotic rim (white arrow) in the epiphysis of the distal femur. After gadolinium contrast administration (coronal T1–Fat–Sat WI, **(b)** the central part of the lesion is non-enhancing whereas there is subtle peripheral rim enhancement (white arrow) with moderate enhancement of the surrounding bone marrow edema.

Although traditionally the growth plate has been considered a barrier for epiphyseal extension of the infectious focus in children because of the specific age-dependent vascularization (Figures 3a–b), this barrier has been shown permeable on magnetic resonance imaging (MRI), as this technique is more sensitive to demonstrate subtle marrow changes as an early sign of infectious spread across the growth plate (Figures 3c and 4).

In rare scenarios, the infection spreads predominantly by the circulus articuli vasculosus of Hunter supplying the epiphysis (Figure 4). This may explain the rare occurrence of epiphyseal childhood osteomyelitis [6].

## Imaging Findings

Imaging findings (Figures 5, 6, 7, 8, 9, 10, 11, 12, 13) depend on the clinical presentation of OM and the age of the patient.

**Figure 5:**
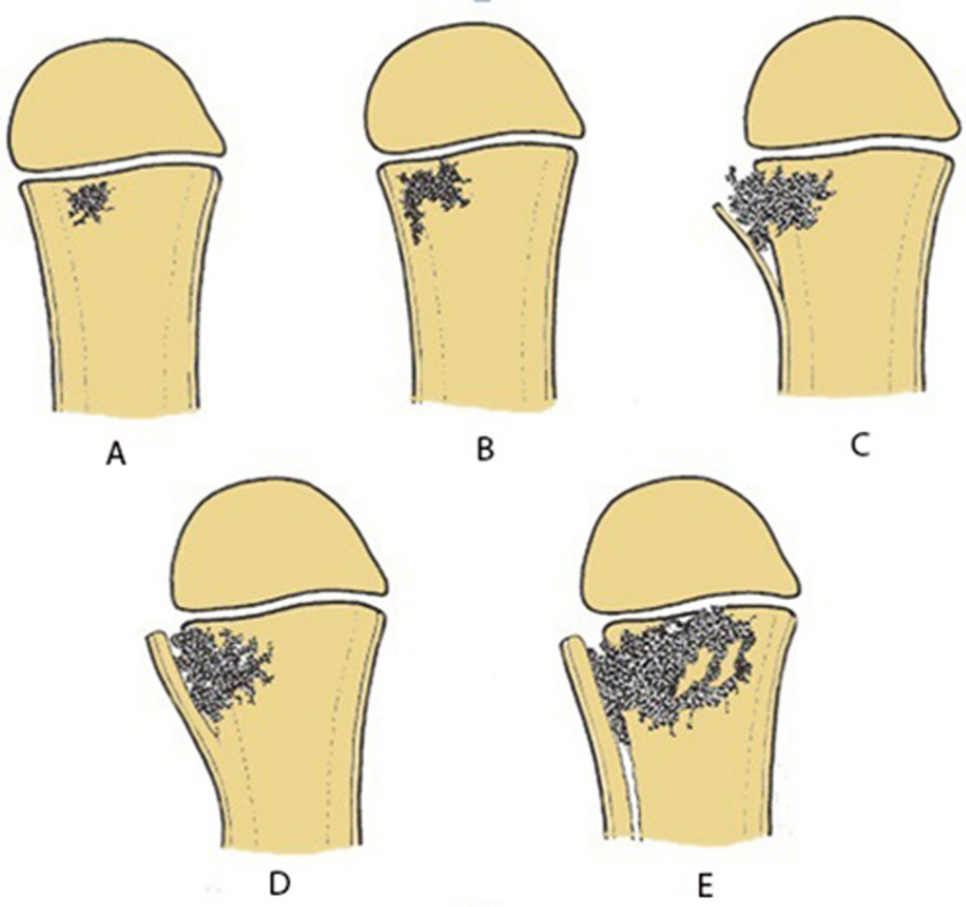
Schematic drawing of the consecutive events of acute osteomyelitis. **(a)** Initial metaphyseal focus. **(b)** Lateral spread to the cortex. **(c)** Cortical penetration and periosteal elevation. **(d)** Formation of thick involucrum. **(e)** Further expansion metaphyseal focus with extensive involucrum.

**Figure 6:**
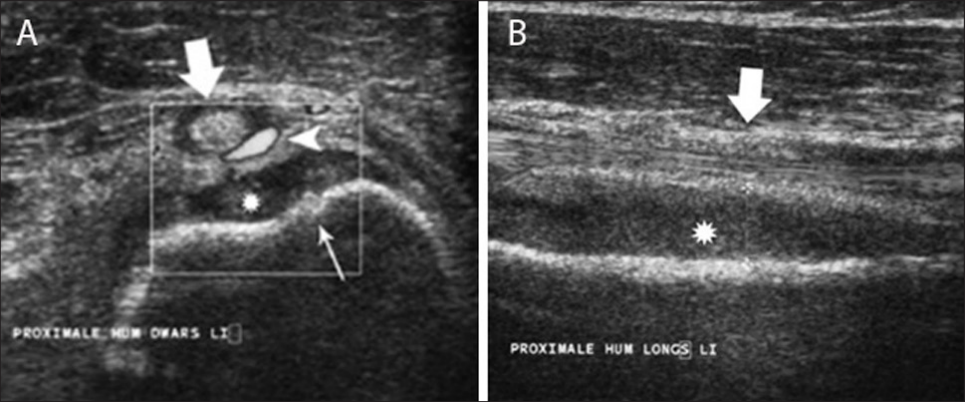
Acute childhood osteomyelitis of the proximal humerus on ultrasound. Transverse **(a)** and longitudinal **(b)** ultrasound images of the proximal humerus. Note focal thinning of the humeral cortex (thin white arrow) on the axial images in keeping with a cortical penetration of the infection causing subperiosteal pus collection (asterisk). There is also increased Doppler signal (white arrow head) within the synovium of the long head of the biceps tendon (large white arrow).

**Figure 7:**
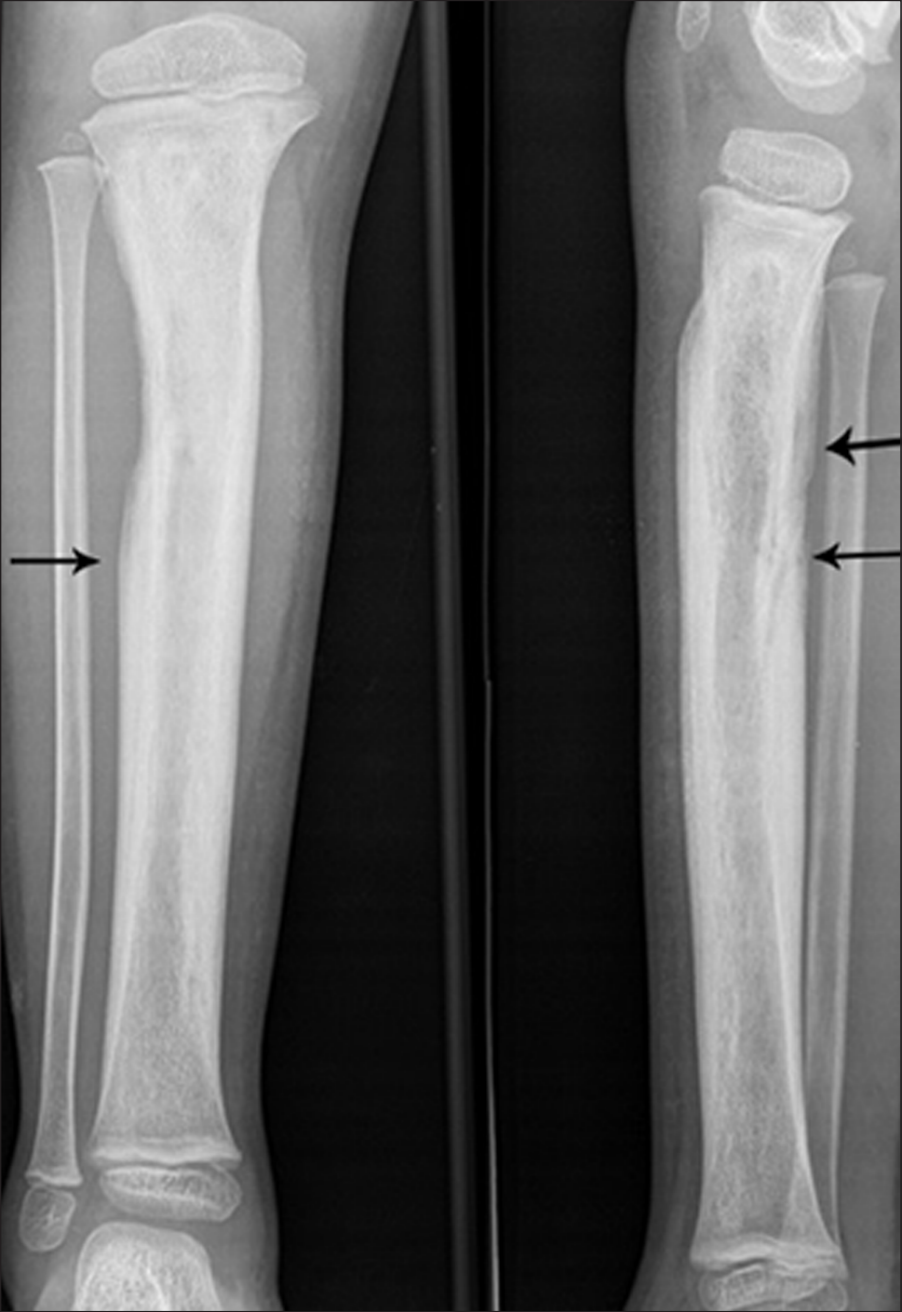
Extensive involucrum on plain radiography. Anteroposterior and lateral plain radiography showing extensive involucrum (arrow) at the tibial diaphysis (black arrows).

**Figure 8:**
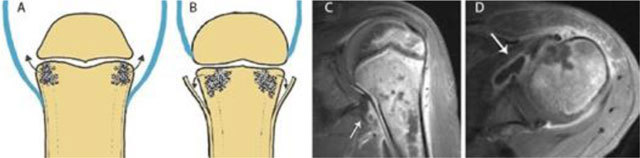
Joint contamination in osteomyelitis. Schematic drawings **(a)** and **(b)**, coronal **(c)** and axial **(d)** T1–WI Fat–Sat after gadolinium contrast administration. **(a)** shows a joint in which the joint capsule (blue) is attaching underneath the growth plate. This intra-articular location of the growth plate may lead to rapid spread of infection into the adjacent joint. **(b)** shows a joint in which the joint capsule (blue) is attaching above the growth plate. An extra-articularly located growth plate protects against early joint contamination. **(c, d)**. Example on MRI of a patient with rapid spread of the infectious focus to the adjacent left shoulder joint. Because the joint capsule of the shoulder insert below the growth plate, metaphyseal osteomyelitis may easily spread through the medial cortex directly into the joint resulting in synovial enhancement (white arrow).

**Figure 9:**
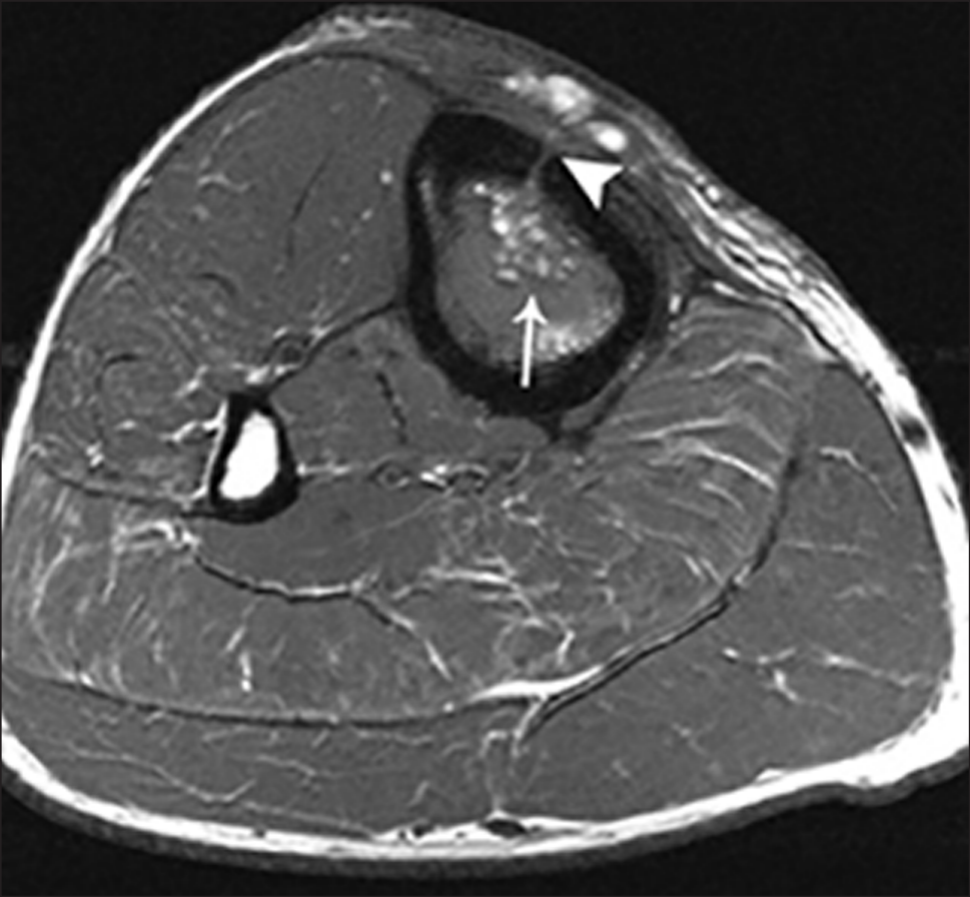
Fat globule sign on T1–WI. Axial T1–WI showing fat globules (white arrow) within the bone marrow edema of the tibia. In addition, there is a cortical defect – also known as the cloaca – perforating the ventromedial cortex of the tibia (white arrowhead).

**Figure 10:**
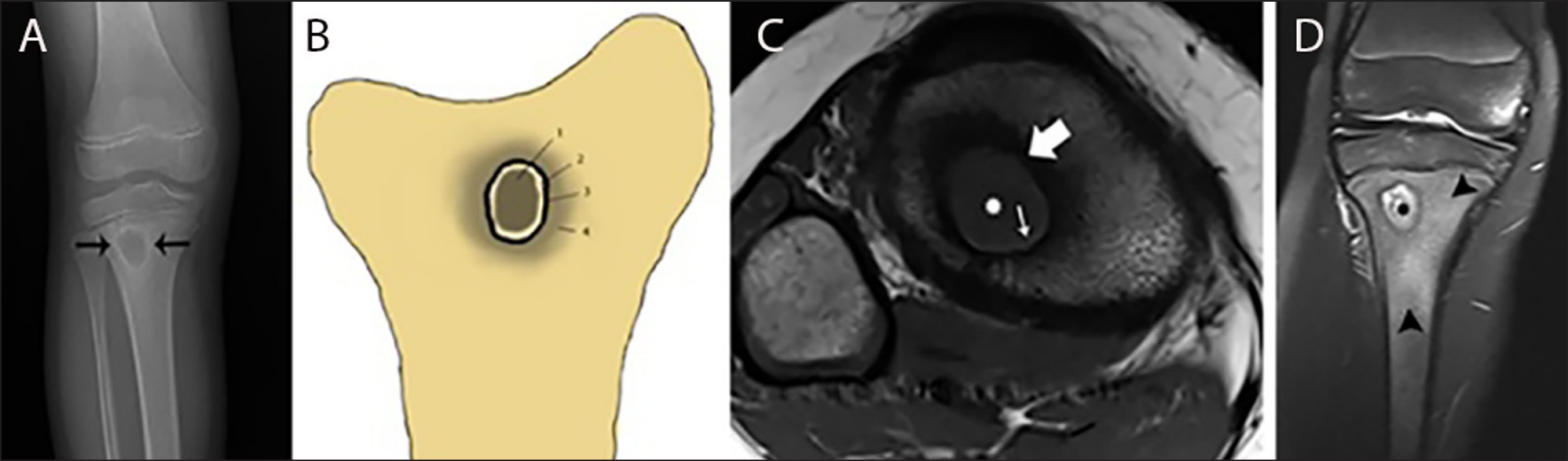
Typical Brodie abscess in subacute osteomyelitis of the tibia. Plain radiograph **(a)** Schematic drawing **(b)** Axial T1–WI **(c)** and coronal Fat–Sat T2–WI **(d)** Plain radiograph **(a)** shows a focal area of metaphyseal osteolysis with a peripheral rim of reactive sclerosis (black arrows). **(b)** shows a the different layers of Brodie abscess on T1–WI with a the pathognomonic penumbra sign on axial T1–WI **(c)** and a pus-filled collection on coronal Fat–Sat T2–WI **(d)**. Central pus of intermediate to low SI on T1–WI (white asterisks on c) and high SI on T2–WI (black asterisks on d). Internal abscess wall consisting of granulation tissue of high SI on T1–WI (penumbra sign) (white small arrow on c) and intermediate SI on T2–WI. External ring of reactional sclerosis of low SI on both T1–WI (white large arrow on c) and T2–WI. Peripheral bone marrow edema of intermediate to low SI on T1–WI and high SI on T2–WI (black arrowheads on d).

**Figure 11:**
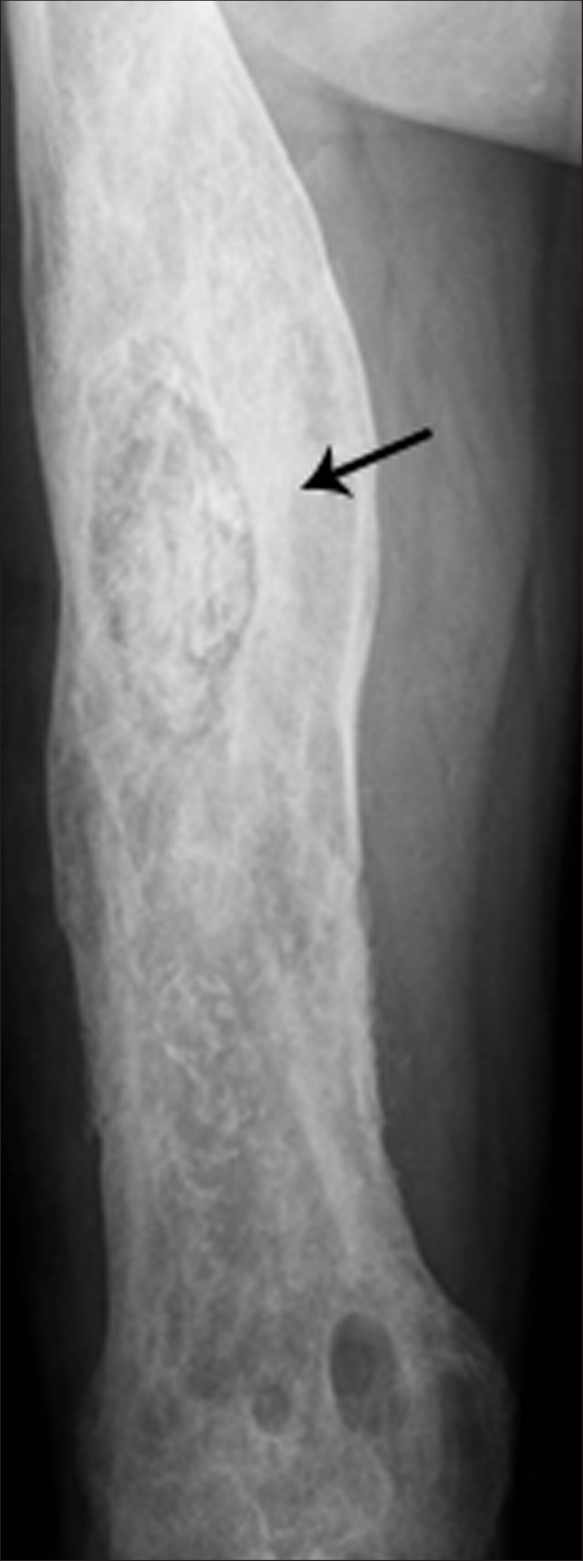
Chronic osteomyelitis on plain radiograph of the femur. Plain radiograph shows diffuse inhomogeneous osteosclerosis of the right femur with a focal area of increased opacity representing necrotic bone or sequestrum (black arrow). Plain films are often difficult to interpret because of superposition of viable and necrotic bone each with a different radiopacity.

**Figure 12:**
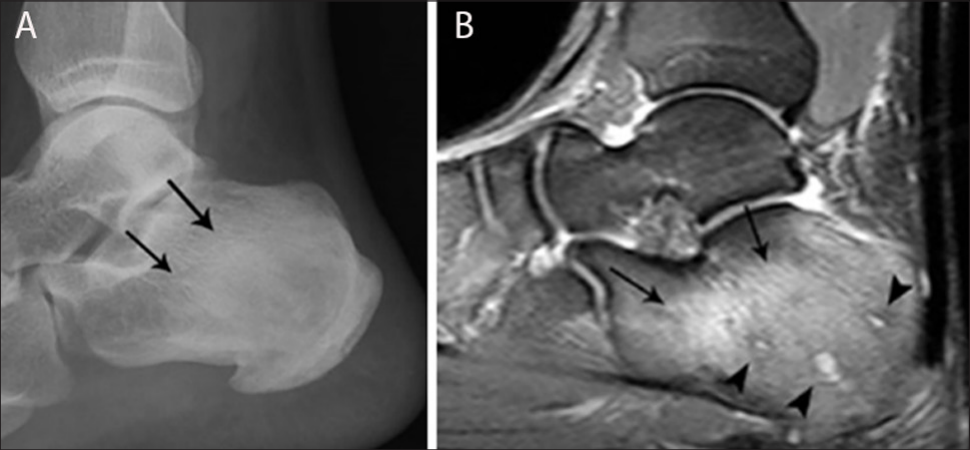
Chronic osteomyelitis of the calcaneus. Plain radiograph **(a)** and sagittal Fat–Sat T2–WI **(b)**. Plain radiograph **(a)** shows inhomogeneous sclerosis (black arrows) in the calcaneus. Bone marrow edema (black arrows) is seen on MRI imaging **(b)**, representing active infection. Note also the presence of small micro-abscesses (black arrowheads).

**Figure 13:**
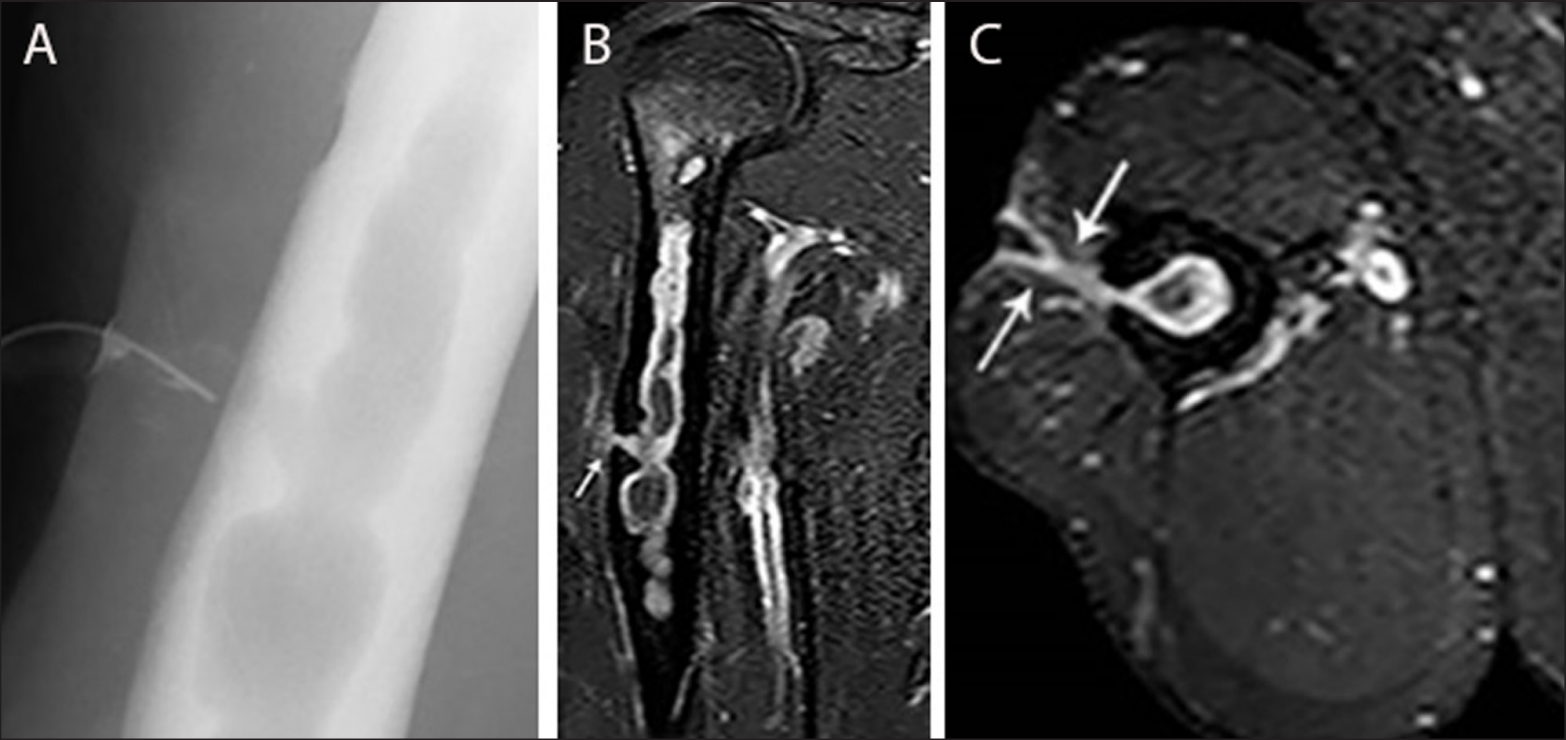
Chronic osteomyelitis of the right humerus with fistula formation. Plain radiograph (sinography) **(a)**, coronal **(b)** and axial **(c)** Fat–Sat T1–WI after gadolinium contrast administration. The sinography **(a)** shows an intramedullary well defined lytic lesion with scalloping of the cortex. Note the presence of a catheter in the sinus. There is enhancement of the wall of the intra-osseous abscess and the wall of the fistula (white arrow) **(b, c)**. Image courtesy Dr. H. Declercq, Dendermonde.

Consecutive changes occurring in *acute OM* are summarized in Figures 3, 4, 5, 6, 7, 8, 9.

Plain radiography is not sensitive for the assessment of the osseous extent of the lesion in the first ten days after onset of infection. Nonspecific soft tissue swelling may be seen. Bony changes such as periosteal thickening, loss of trabecular architecture, osteopenia and osteolytic destruction are seen after one week at the earliest. However, plain radiography is useful as a baseline study for further follow-up and for differential diagnosis purposes.

Ultrasound (US) allows easy comparison of both sides. It is an accurate and quick tool to detect subperiosteal spread in acute OM particularly in children because of loose attachment of the periosteum. Visualization of periosteal elevation, fluid collections or abscesses are signs suggestive of osteomyelitis. A soft tissue abscess presents as a hypo-echogenic collection to normal muscle with a peripheral vascularized rim of increased Power Doppler signal. US detects these signs earlier than standard radiography and allows US-guided biopsy and/or aspiration (Figure 6) [7].

Magnetic Resonance Imaging is the modality of choice for early detection of acute OM. Bone marrow changes are detected within three to five days after disease onset on Fat–Sat T2-weighted imaging (WI). OM can spread to the joint depending on the position of the joint capsule compared to the growth plate. An extra-articular location of the growth plate does not predispose to joint infection, whereas intra-articular position of the growth plate may cause rapid spread to the adjacent joint (Figure 8). Soft tissue extension, joint effusion, abscess formation and sinus tracts are accurately visualized. A pathognomonic sign for acute osteomyelitis is the presence of intramedullar fat globules on T1–WI. Islands of fat are released by necrotic lipocytes resulting in high signal intensity (SI) on T1–WI (Figure 9) [8,9,10].

Subacute osteomyelitis is characterized by the presence of Brodie’s abscess at the metaphysis. It typically presents as an oval radiolucent lesion with peripheral sclerosis within the metaphysis of the long bones on plain radiography (Figure 10). The shape of a Brodie’s abscess along the longitudinal axis may be explained by effect of gravity. More rarely, Brodie’s abscess may be located in the short and flat bones as well. The presentation of a Brodie’s abscess on MRI (Figure 10) is characterized by the penumbra sign consisting of a subtle peripheral rim of high signal on T1–WI around the pus collection in the bone. The high SI is probably due to granulation tissue in the abscess wall with lipid-laden macrophages (Figure 10) [10]. After administration of intravenous gadolinium contrast, this thin vascularized rim enhances [11,12].

Inhomogeneous osteosclerosis and/or sequestrum formation (necrotic bone) is characteristic for chronic osteomyelitis on plain radiography. A sequestrum represents a segment of necrotic bone that is separated from the living bone by granulation tissue and bone resorption. It is typically denser than the living bone [13]. In some cases, a layer of new periosteal bone or involucrum is formed around the necrotic bone (Figures 7, 11, 12). On MRI, a sequestrum is hypo-intense on all pulse sequences. Gadolinium contrast administration may reveal a cloaca (opening in the involucrum) through which pus, granulation tissue and sequestra can be discharged. In addition, enhancement of sinus tracts tracking from the bone to the skin surface is well demonstrated on contrast enhanced MRI (Figure 13) [15].

Bone scintigraphy is able to detect osteomyelitis with high sensitivity. In the delayed phase an increased activity in the affected bone is seen, uptake in the surrounding bone and/or soft tissue may be present as well. The specificity is, however, low. Multiplicity is easily detected by this method [14].

Computed Tomography (CT) is useful in evaluating chronic osteomyelitis in areas with a complex anatomy. CT may provide information regarding the presence of sequestra, cloaca, cortical destruction and the thickness of the involucrum. Particularly in the assessment of sequestrum formation, CT is more accurate than plain radiography and MRI. In addition, it is a useful technique for imaging-guided needle biopsy and aspiration material for microbiology [14]. However, due to radiation dose, use of CT should be carefully outweighed in children.

Garré’s sclerosing osteomyelitis is a specific subtype typically affecting the mandible but can also be seen in the long bones. Children and young adults are mainly affected. The etiology remains unclear, as the cultures are generally negative an underlying viral infection is suspected [16]. Patients present with pain, swelling and trismus if the mandible is affected. Imaging shows marked thickening of the periosteum with peripheral reactive bone formation. Treatment of mandibular involvement consists of surgical excision of the causative tooth [17].

## Special Subtypes of Osteomyelitis: Chronic Recurrent Multifocal Osteomyelitis (CRMO) and SAPHO

### Definition and pathogenesis

CRMO is defined as an auto-immune disease with recurrent inflammatory lesions of the bone that typically affects children [18]. It typically affects the medial portion of the clavicle and metaphyses of the long bones, but other locations may be involved as well. Patients present with progressive swelling and pain of the affected site.

**Figure 14:**
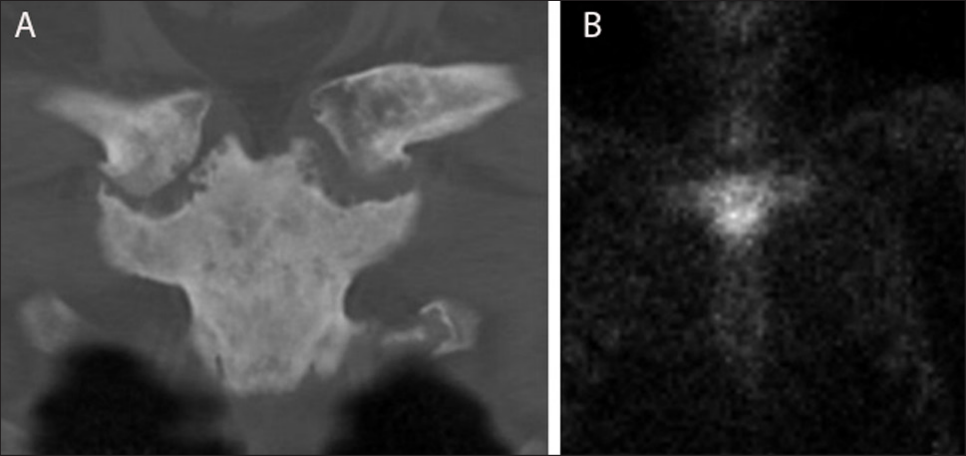
Typical bullhead sign in SAPHO of the sternoclavicular joint. CT (coronal reformatted image) **(a)** shows sclerosis of the manubrium sterni and medial clavicles and erosions of the sternoclavicular joints. Note a typical bullhead sign on scintigraphy **(b)**.

The combination of synovitis, acne, pustulosis, hyperostosis and osteitis is designated as SAPHO and is seen as the adult counterpart of CRMO. It represents an inflammatory disorder of unknown etiology in which osteitis is a key symptom. The axial skeleton is preferentially affected with predominant involvement of the sternoclavicular joints (bilateral sternoclavicular joint edema) (Figure 14) and the spine. Sacro-iliac joints are not commonly involved.

### Imaging

**Figure 15:**
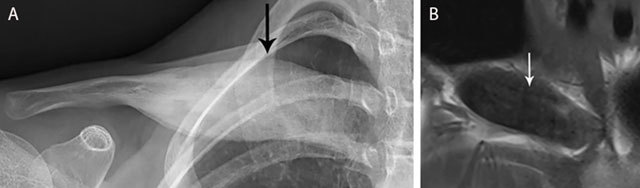
CRMO of the medial clavicle. Plain radiograph **(a)** shows an expansile lesion (black arrow) of the medial clavicle with extensive bone sclerosis and a solid periosteal reaction. The lesion is of hypo-intense signal on coronal T1–WI **(b)**. Note osseous expansion of the lesion beyond the original cortex of the clavicle (white arrow).

Plain radiography in CRMO initially shows osteolytic lesions, sometimes associated with periosteal reaction having a so-called “onion ring” appearance (Figure 15a). In later stages, there is progressive sclerosis of the bone. CRMO of the clavicle typically affects the medial side. Radiographically active lesions may be clinically silent and there is no correlation between the symptoms and activity on imaging [19].

In SAPHO, the affected joints show sclerosis and erosions of the joint facets which are all signs of arthritis. Hyperostosis and enthesopathy are visualized [20].

CT is able to detect and assess the extent of these bony changes with precision [19].

MRI shows bone marrow edema in acute CRMO lesions with a hypo-intense SI on T1–WI and hyperintense SI on T2–WI (Figure 15b). Transphyseal invasion may be visualized. Progressive sclerosis results in hypo-intense signal on both T1– and T2–WI and may be seen in subacute and chronic lesions. [19]. MRI is the preferred imaging technique for early detection of SAPHO. Bone marrow edema is readily visualized on T2–WI allowing to differentiate acute and chronic lesions.

Of note, according to other authors CRMO and SAPHO belong to the spectrum of spondylarthropathy rather than being an infection. Further discussion of these entities is therefore beyond the scope of this pictorial review.

## Conclusion

The variable imaging appearance of osteomyelitis may be explained by the different pathogenic mechanisms involved in the spread of the infection and by the age-related vascularization of bone. Standard radiography is still the baseline examination for follow-up and differential diagnosis. Ultrasound is the preferred modality in case of suspicion of acute osteomyelitis in children or concomitant septic arthritis. US-guided biopsies and/or aspiration are safely and easily performed. CT may be useful in the evaluation of chronic osteomyelitis, particularly in areas with a complex anatomy. CT may provide information regarding the presence of sequestra, cloaca, cortical destruction and reactive involucrum formation. In addition, it is used for imaging-guided biopsy and aspiration of infectious material for microbiological examination. MRI is the preferred modality for early detection of osteomyelitis. The fat globule sign on T1–WI is pathognomonic for acute osteomyelitis whilst the penumbra sign is pathognomonic for a Brodie abscess in subacute osteomyelitis. A combination of T1– and Fat–Sat T2–WI and gadolinium enhanced imaging is mandatory.
